# Accessories in clear aligner therapy: Laypeople’s expectations for comfort and satisfaction

**DOI:** 10.34172/joddd.40793

**Published:** 2024-06-24

**Authors:** Jussimar Scheffer Castilhos, Gil Guilherme Gasparello, Sergio Luiz Mota-Júnior, Giovani Ceron Hartmann, Luiz Fernando Iyda Miyagusuku, Matheus Melo Pithon, Orlando Motohiro Tanaka

**Affiliations:** ^1^Department of Orthodontics, Medicine and Life Sciences School, Pontifícia Universidade Católica do Paraná, Curitiba, Brazil; ^2^Orthodontist, Private Practice, Juiz de Fora Federal University, Juiz de Fora, Minas Gerais, Brazil; ^3^Undergraduate Student in Dentistry, Medicine and Life Sciences School, Pontifícia Universidade Católica do Paraná, Curitiba, Brazil; ^4^Department of Orthodontics, Southwest Bahia State University – UESB, Jequié, Bahia, Brazil

**Keywords:** Clear aligner, Orthodontics, Patient preferences, Visual perception

## Abstract

**Background.:**

This study explored the layperson’s perception of comfort, satisfaction, and willingness to use various accessories in clear aligner therapy.

**Methods.:**

A total of 267 people analyzed standardized intraoral photographs of a female model in orthodontic treatment using: 1) only clear aligner (CA), the control group; 2) clear aligner+attachments (AT); 3) clear aligners+Cl II elastics (EL); 4) clear aligner+hybrid treatment with esthetic braces (HEB); 5) Clear aligner+hybrid treatment with metallic braces (HMB); 6) clear aligner+mini-implants (MI); 7) clear aligner+mini-implants and elastics for intrusion (MIE). In addition, a social media questionnaire was distributed to assess the willingness to undergo orthodontic treatment with various accessories.

**Results.:**

There was a significant difference between CA and all the other groups (*P*<0.001), with CA being considered more comfortable and providing greater satisfaction compared to other accessories. Moreover, AT showed a significant difference in reducing treatment time compared to other groups.

**Conclusion.:**

The CA was the most comfortable, exhibiting a higher satisfaction rate and a greater willingness to use it. The AT therapy was perceived as more comfortable and was associated with higher satisfaction and a greater likelihood of use, especially if it resulted in reduced treatment time. On the other hand, the participants reported that the HMB, MI, and MIE accessories were less comfortable.

## Introduction

 The demand for aesthetic treatments among patients has significantly increased, necessitating orthodontic appliances that prioritize aesthetics throughout the treatment process. In response to this demand, manufacturers have developed systems specifically designed to appeal to patients, with the primary objective of minimizing the visibility of the appliance.^1^

 Recently, patients have been highly involved in selecting orthodontic techniques and have shown an active interest, higher expectations, and an emphasis on the quality of life elements in treatment.^2^ Simultaneously, modern orthodontics strives to offer patients a comfortable and pleasant treatment journey.^3^

 Since aligners have evolved, many brands have begun integrating auxiliary systems like elastics and mini-implants to increase treatment options.^4^ The thermoplastic materials used, gingival margin design, and the different strategies used to guide orthodontic movements, such as attachments, mini-implants, elastics, and other auxiliary tools, converge in determining the effectiveness of a system of aligners,^5^ directly correlated to the entire resolution of a case being successfully treated.^6,7^

 Many factors are involved in patient satisfaction, which can be considered a mixture of the patient’s beliefs, the perceived impact of treatment on quality of life, and the perceived quality of the service provided by the dental team.^8^ Moreover, discomfort during treatment may influence the level of satisfaction.^9^

 The standard way of assessing patient perspective and satisfaction relies on surveys and questionnaires.^8^ A previous study showed that nearly two-thirds of young adults would reject orthodontic treatment if it involved treatment with visible appliances.^10^ Visible options were not only seen as less attractive but also led to the user’s assumption that they had less favorable characteristics.^11^

 As a result, clear aligners have quickly become synonymous with aesthetics for most patients, and this is how the product is currently marketed. However, contrary to marketing, clear aligners primarily require accessories for improved treatment, such as attachments, elastics, cuts, hybrid treatment, mini-implants, and other options. Additionally, studies have revealed a general preference among laypeople for clear aligners with minimal accessories and ceramic brackets over clear aligners with multiple attachments.^12,13^

 The present study investigated patients’ willingness to undergo orthodontic treatment with clear aligners and their attitudes toward orthodontic movement accessories. The null hypothesis stated that there are no significant differences in terms of comfort, satisfaction, and willingness to use various orthodontic treatment methods, including only clear aligner, clear aligner + attachments, clear aligners + Cl II elastics, clear aligner + hybrid treatment with esthetic braces, clear aligner + hybrid treatment with metallic braces, clear aligner + mini-implants, and clear aligner + mini-implants and elastics for intrusion.

## Methods

 This cross-sectional study was approved by the university’s ethics committee (approval number 2,235,302). All the participants provided informed consent before completing the online survey. The study aimed to include individuals > 18 years of age from diverse backgrounds, regardless of ethnicity, education, or socioeconomic status. Laypeople from two cities in different regions were recruited for the study, and the raters had no prior experience with orthodontic appliances. Participants were approached in the city center and invited to participate in the study.

 A calculation was performed using an infinite population, a 95% confidence level, and a 6% margin of error to determine the appropriate sample size for the research study. Based on this formula, the sample size was determined at 267 individuals. This ensured that the study would have a sufficient sample size to produce statistically significant results.


(1) $$sample\;size=\left[z2*p\left(1-p\right)\right]/e2/1+\left[z2*p\left(1-p\right)\right]/e2*N]$$

###  Images standardization and questionnaire construction 

 Frontal and lateral intraoral photographs and a smiling frontal view were captured using a digital camera (Rebel XTI; Canon, Tokyo, Japan) in a studio setting with appropriate lighting. The photographs were taken with accessories installed, except for the mini-implants group, which were added using Photoshop (Adobe Systems Inc., San Jose, California). All the images were analyzed, selected, and standardized for consistency. To avoid any asymmetries, the images were mirrored, and any dark restorations were removed to ensure accurate analysis.

**Figure 1:**
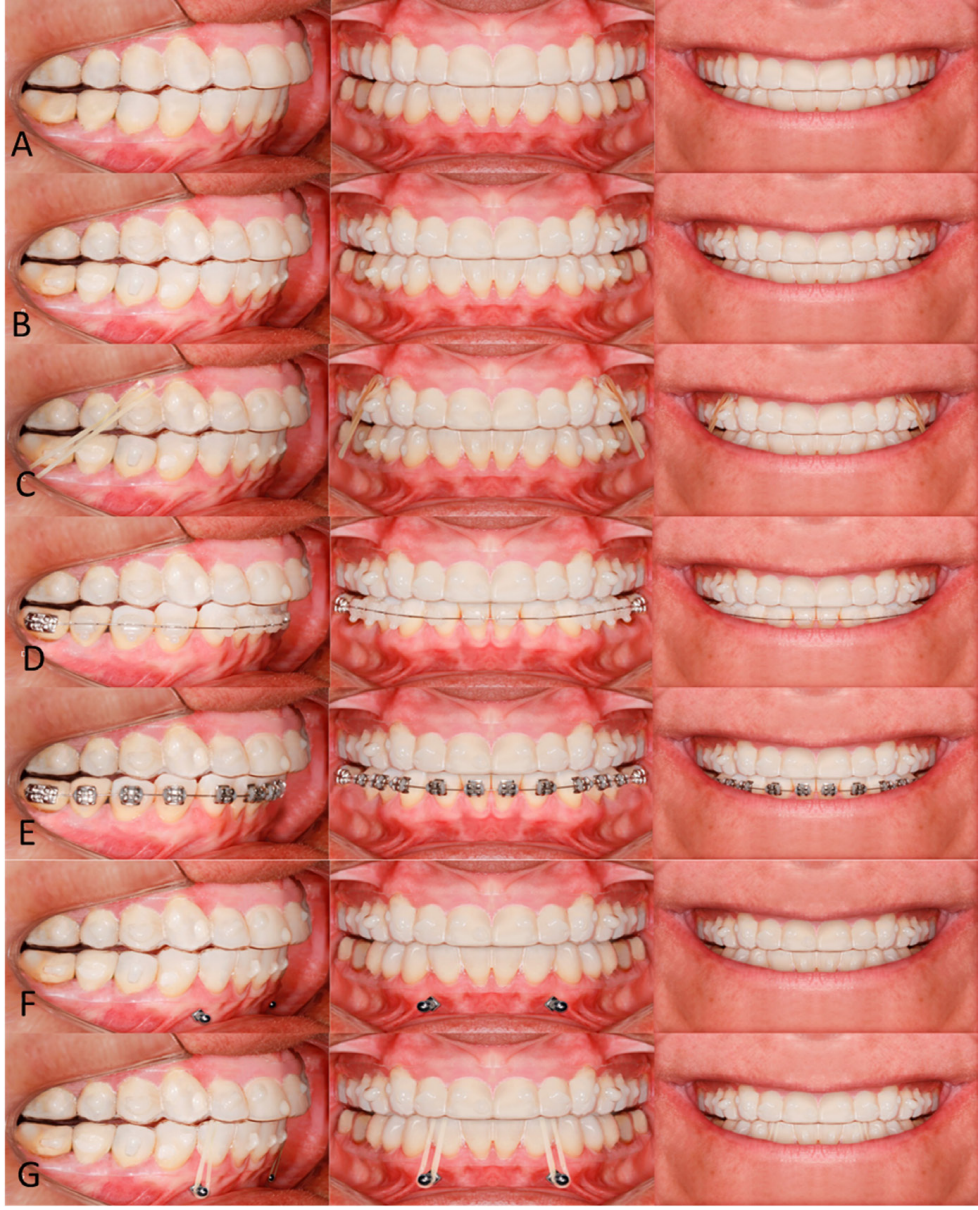
Images evaluated A. Only clear aligner (CA). B. Attachments (AT). C. Cl II elastic (EL). D. Hybrid esthetic braces (HEB). E. Hybrid metallic braces (HMB). F. Mini-implants. G. Mini-implant + elastic

 The accessory groups evaluated in this research were: 1) only clear aligner (CA), the control group; 2) clear aligner + attachments (AT); 3) clear aligners + Cl II elastics (EL); 4) clear aligner + hybrid treatment with esthetic braces (HEB); 5) Clear aligner + hybrid treatment with metallic braces (HMB); 6) clear aligner + mini-implants (MI); 7) clear aligner + mini-implants and elastics for intrusion (MIE). The composition of the evaluated images is presented in Figure 1.

 Questions from three separate research studies about willingness to undergo the procedure with clear aligners and accessories were used to construct the questionnaire. After a discussion, three questions were selected and asked after each image: (1) Would you feel comfortable going through this procedure? (Yes, No); (2) Would you be satisfied if this procedure was required for your treatment? (Yes, No); (3) If this procedure would reduce treatment time, I would use this accessory (analyzed through a visual analog scale [VAS]). (“0”: I would never use it; “10”: I would willingly use it.)

###  Reliability 

 To ensure the questionnaire’s validity and reliability, it was administered twice during the preliminary data collection stage, with a 20-day interval between each administration. Thirty subjects were included in the questionnaire’s first (test) and second (retest) administrations, and these subjects were not included in the final sample. The purpose of this testing was to evaluate the consistency of the questionnaire and ensure that it was an effective tool for collecting accurate data.

###  Questionnaire application and participants’ backgrounds

 For data collection, the questionnaire link was distributed to the participants via social media (Facebook, Instagram, LinkedIn, and WhatsApp) using the Qualtrics digital platform (Salt Lake City, UT, USA). Participants could access the questionnaire digitally using a computer, smartphone, or tablet. The questionnaire was available only in Portuguese. The response was validated if the participant stated that he was older than 18, accepted the online terms, and answered all the questions. The study included 267 participants with a mean age of 27.79 years (range: 18‒65), 94 with incomplete higher education, and 173 college graduates or higher out of 100 males (mean age: 27.40 years, range: 18‒52), with 72 graduates, 28 undergraduates, 167 females (mean age: 28.03, range: 18‒65), 101 graduates, and 66 undergraduates.

###  Data analysis

 The data were collated through Microsoft Excel in an electronic database (Microsoft, Inc., Redmond, WA, USA). SPSS 25 was used for the statistical analysis (IBM, Armonk, USA). The intraclass correlation (Cronbach’s alpha coefficient) was used to measure internal consistency. The mean and standard deviation of the numerical variables and the total count and percentage of the qualitative variables were calculated using descriptive statistics. To compare the dependent variables between the groups, one-way ANOVA and chi-squared tests were used with a significance level of 5% (*P* < 0.05).

## Results

**Figure 2:**
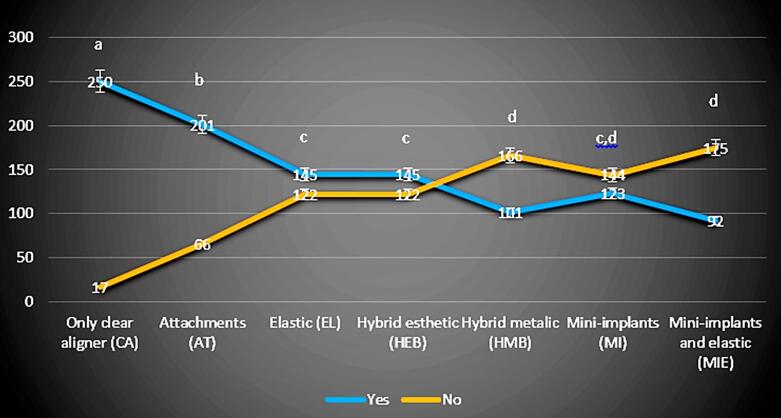
Chart regarding “Would you feel comfortable going through this procedure?”

 The questionnaire showed satisfactory internal consistency (Cronbach’s alpha = 0.819). A total of 267 participants were included. No sexual dimorphism was found (*P* > 0.05), nor was it found that the educational background influenced the evaluators’ perception (*P* > 0.05). The results for question (1), “Would you feel comfortable going through this procedure?” showed a significant difference between CA and all the other groups (*P* < 0.001), with CA being considered more comfortable compared to other accessories. A similar significant difference was found for AT and all the other groups (*P* < 0.001), which was perceived as less comfortable than CA but also considered significantly more comfortable than EL, HEB, HMB, MI, and MIE (Figure 2).

**Figure 3:**
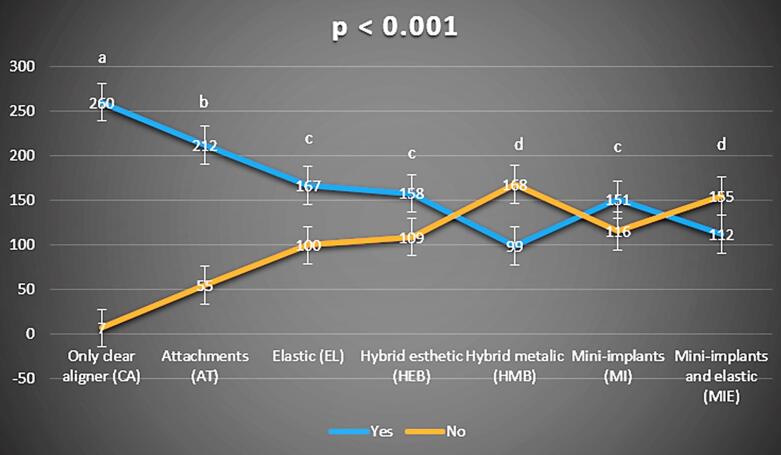
Chart regarding “Would you be satisfied if this procedure were necessary for your treatment?”

 Regarding the question (2), “Would you be satisfied if this procedure would be necessary for your treatment?” (Yes, No)” showed a significant difference between CA and all the other groups (*P* < 0.001), in which CA showed a higher satisfaction ratio than all the other accessories. AT recorded a significant difference for all the other groups (*P* < 0.001). AT was correlated with less satisfaction than CA but was also considered statistically correlated with better satisfaction than EL, HEB, HMB, MI, and MIE. The HMB and MIE were associated with significantly lower levels of satisfaction. HEB was associated with a higher satisfaction ratio than HMB. A higher satisfaction ratio was also found when comparing MI and MIE, with a higher satisfaction ratio in the MI group (Figure 3).

**Figure 4:**
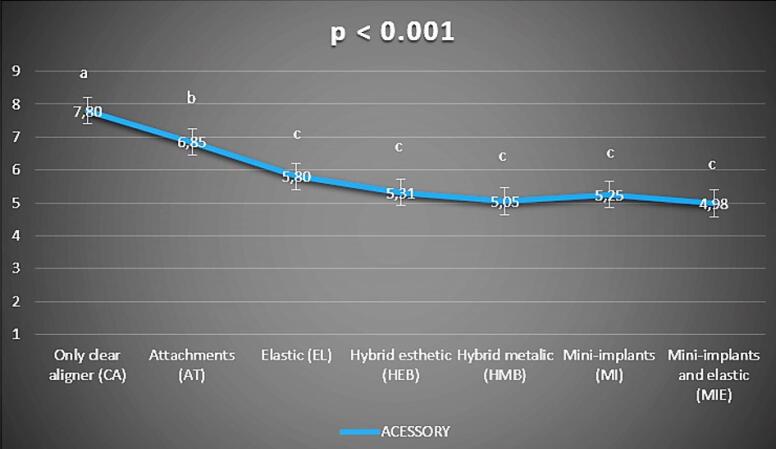
Chart regarding “If this procedure would reduce treatment time, I would use this accessory.”

 The results for question (3), “If this procedure reduced treatment time, I would use this accessory” (analyzed through a visual analog scale, VAS) (“0” I would never use it; “10” I would willingly use it) showed significant differences between CA and all the other accessories (*P* < 0.05) and scored higher points for VAS in the respondents’ willingness to use it. Furthermore, the AT group showed a significant difference from all the other groups, scoring significantly lower points than CA (CA mean: 7.80, AT mean: 6.85, *P* = 0.011) but significantly higher points than EL, HEB, HMB, MI, and MIE (*P* > 0.05). The group’s EL, HEB, HMB, MI, and MIE did not show a significant difference between them (Table 1, Figure 4).

**Table 1:** ANOVA regarding willingness to use if treatment time is reduced and post hoc tests (N = 267)

**Accessory**	**Mean**	**Standard deviation **		**Post hoc test**			
			***P*** ** value for ANOVA**	**Accessory comparison**			***P*** ** value post- Hoc**
Only clear aligner	7.80	3.08	< 0.001	Attachments	0.951^*^	0.277	0.011
				Elastic	1.996^*^	0.282	0.000
				Hybrid esthetic	2.487^*^	0.276	0.000
				Hybrid metallic	2.745^*^	0.270	0.000
				Mini-implants	2.551^*^	0.294	0.000
				Mini-implants and elastic	2.816^*^	0.282	0.000
Attachments	6.85	3.31	< 0.001	Only clear aligner	-0.951^*^	0.277	0.011
				Elastic	1.045^*^	0.291	0.007
				Hybrid esthetic	1.536^*^	0.285	0.000
				Hybrid metallic	1.794^*^	0.281	0.000
				Mini-implants	1.599^*^	0.303	0.000
				Mini-implants and elastic	1.865^*^	0.292	0.000
Elastic	5.81	3.42	< 0.001	Only clear aligner	-1.996^*^	0.282	0.000
				Attachments	-1.045^*^	0.291	0.007
				Hybrid esthetic	0.491	0.290	0.622
				Hybrid metallic	0.749	0.285	0.120
				Mini-implants	0.554	0.307	0.546
				Mini-implants and elastic	0.820	0.296	0.084
Hybrid esthetic	5.31	3.28	< 0.001	Only clear aligner	-2.487^*^	0.276	0.000
				Attachments	-1.536^*^	0.285	0.000
				Elastic	-0.491	0.290	0.622
				Hybrid metallic	0.258	0.279	0.968
				Mini-implants	0.064	0.302	1.000
				Mini-implants and elastic	0.330	0.290	0.917
Hybrid metallic	5.06	3.17	< 0.001	Only clear aligner	-2.745^*^	0.270	0.000
				Attachments	-1.794^*^	0.281	0.000
				Elastic	-0.749	0.285	0.120
				Hybrid esthetic	-0.258	0.279	0.968
				Mini-implants	-0.195	0.297	0.995
				Mini-implants and elastic	0.071	0.286	1.000
Mini-implants	5.25	3.68	< 0.001	Only clear aligner	-2.551^*^	0.294	0.000
				Attachments	-1.599^*^	0.303	0.000
				Elastic	-0.554	0.307	0.546
				Hybrid esthetic	-0.064	0.302	1.000
				Hybrid metallic	0.195	0.297	0.995
				Mini-implants and elastic	0.266	0.308	0.978
Mini-implants and elastic	4.99	3.42	< 0.001	Only clear aligner	-2.816^*^	0.282	0.000
				Attachments	-1.865^*^	0.292	0.000
				Elastic	-0.820	0.296	0.084
				Hybrid esthetic	-0.330	0.290	0.917
				Hybrid metallic	-0.071	0.286	1.000
				Mini-implants	-0.266	0.308	0.978

Statistical Significance when *P* < 0.05

## Discussion

 Clear aligner mechanotherapy has emerged as a key cosmetic alternative^14^ since malocclusion has been shown to affect social life,^15^ and appliances have an essential role in patients’ esthetic perception.^12^ It has been suggested that clear aligners are less effective than conventional fixed appliances for some tooth movements. Because of these limitations, accessories must be used to increase and optimize those movements.^16^ In this context, this study evaluated the comfort, satisfaction, and willingness of laypeople to use clear aligners in combination with additional accessories or hybrid treatment.

 Attachments are force transducers used to enhance biomechanics^17,18^ and may be described as a protrusion of composite resin material polymerized onto the tooth surface.^19^ Although their presence might be perceived by laypeople, a study regarding eye-tracking found that laypeople perceived the accessory. There is a general inclination for clear aligners without attachments and esthetic brackets over clear aligners with multiple attachments,^12^ which corroborates our study, since the raters felt more comfortable (250 people [93.6%]) said “yes.” In contrast, 17 (6.4%) said “no” about the comfort seen in clear aligner usage (*P* < 0.001), satisfied (260 [97.4%]) people said “yes,” while 7 (2.6%) said “no” about the satisfaction seen in clear aligner usage (*P* < 0.001) and demonstrated willingness (7.8 for VAS score, with *P* < 0.001 for higher VAS, compared with all the others accessories) to use only aligners.

 In line with findings from Thai et al,^12^ only clear aligners were the most accepted by raters in the present study. However, it is worth noting that the methodology differed between studies. Thai et al used photographs with esthetic fixed appliances in the upper and lower arches to compare with clear aligners with few or many attachments, while in the present study, apart from the control group, all photographs depicted clear aligners with many attachments in the upper arch. This difference in methodology could have contributed to the varying results observed.

 Some aligner companies challenge the use of attachments and advocate for their nearly complete omission and alternative instruments for movement control. The indications for using aligners for orthodontic therapies—even those of a certain complexity that the aligners alone would not be able to manage predictably—have undoubtedly been expanded with the inclusion of auxiliary devices like elastics and mini-implants and even mixing traditional and esthetic braces. The significant factors determining the effectiveness and efficiency of the various aligner systems are the thermoplastic material from which the aligners are constructed and the ability to use attachments or alternate movement techniques.^20^ The present study showed that laypeople did not fully accept using extra accessories with clear aligners as a complementary device to achieve better results compared with clear aligners only (*P* < 0.001). This can be explained by the patients’ demands for aesthetic treatments, which aim to reduce the device’s visibility.^21^

 Work-related and professional factors contribute to the interest in less-visible treatment options such as ceramic brackets and lingual or aligner appliances.^22^ Försch et al^13^ described the lingual appliance as the only one that is similar to the aligners in terms of perception. However, patients treated with lingual orthodontic appliances experienced more tongue and earlier pain than with other appliances.^23^ In terms of comfort, this study presented the AT group as more comfortable, with a higher satisfaction ratio and willingness to go through this therapy than HEB and all the other accessories evaluated, except for the CA group.

 The treatment results with clear aligners can be improved using mini-implants^24,25^ and associated elastics.^26^ In this study, the groups with mini-implants (MI, MIE, and HMB) were considered less comfortable. The MIE and HMB groups had the lowest satisfaction ratio, though if necessary and to reduce treatment time, laypeople’s acceptance of MI and MIE showed no statistical difference compared to EL, HEB, and HMB.

 Concerning mild to severe cases, hybrid treatment with braces and CA might be performed; the literature describes some cases.^27,28^ According to one study, orthodontic appliances were acceptable but not as effective as CA in a young population.^29^ Moreover, growing patients between the ages of 8 and 16 already have a strong self-perception regarding smile esthetics. They are amenable to types of orthodontic treatment that involve using aesthetic appliances, which makes hybrid therapy more indicated, in addition to being comfortable.^30^ In the present study, the results showed a significant difference between all the variables evaluated in favor of the CA group regarding HEB and HMB, which could compromise the quality of orthodontic treatment.^31^ As CA becomes increasingly marketed as an “invisible” orthodontic treatment, the findings of the present study underscore the importance of open communication between orthodontists and patients about the need for additional mechanics and accessories to achieve optimal results. Orthodontists should demonstrate greater professionalism and adopt innovative methods to educate patients about CA treatment and its requirements to ensure the best possible outcome.

 This study had some limitations that should be considered. For example, evaluating willingness to undergo a procedure, comfort, and satisfaction are complex features that are difficult to measure solely through images. Therefore, our approach was to gather the participants’ perceptions, and the results should be interpreted with caution, particularly given the limitation of the questionnaire’s distribution to different regions of only one country. Additionally, several additional mechanics, such as enamel reduction, power arms, bite ramps, and so on, may be used with CA that were not included in this study. However, despite these limitations, this study aimed to determine whether patients are willing to undergo orthodontic treatment with clear aligners and whether they accept the need for orthodontic accessories to aid in orthodontic movement.

 Moreover, it is important to highlight that advertisements may confuse patients due to the frequent need for orthodontic movement accessories during treatment. Additionally, this study provides insight into patients’ perceptions of comfort, satisfaction, and willingness to undergo clear aligner therapy, which can aid in developing a more effective treatment plan based on the individual’s specific malocclusion. Furthermore, understanding these patient preferences can improve the relationship and communication between orthodontists and their patients, leading to better treatment outcomes.

## Conclusion

 The control group (CA) emerged as the most comfortable option, displaying a higher satisfaction ratio and greater willingness to use it than all the other evaluated accessories. The attachment therapy (AT) was perceived as more comfortable and demonstrated higher levels of satisfaction and willingness to use, particularly when it resulted in reduced treatment time, in comparison to Cl II elastics (EL), HMB, HEB, MI, and MIE. The patients considered HMB and mini-implants (MI and MIE) less comfortable. Patients found HEB to be more comfortable and associated them with higher satisfaction than HMB.

## Acknowledgments

 We would like to extend our heartfelt gratitude to all the participants whose contributions were invaluable to this study. Additionally, our sincere thanks go to the PIBIC-PUCPR scholarship program for its support throughout our research.

## Competing Interests

 The authors declare that there are no conflicts of interest regarding the publication of this paper.

## Ethical Approval

 The study was approved by the Research Ethics Committee of the University, with the approval code/number 2,235,302.

## Funding

 This research received no specific grant from funding agencies in the public, commercial, or not-for-profit sectors.
